# The significant places of African American adults and their perceived influence on cardiovascular disease risk behaviors

**DOI:** 10.1186/s12889-021-12022-x

**Published:** 2021-11-05

**Authors:** Michelle J. White, Katelyn M. Holliday, Stephanie Hoover, Nicole Robinson-Ezekwe, Giselle Corbie-Smith, Anissa Williams, Kiana Bess, Leah Frerichs

**Affiliations:** 1 Department of Pediatrics, Duke University School of Medicine, DUMC 102376, 2301 Erwin Rd, Durham, NC 27705 USA; 2 Department of Family Medicine and Community Health, Duke University School of Medicine, DUMC 2914, Durham, NC 27710 USA; 3 Center for Health Equity Research, Department of Social Medicine, University of North Carolina at Chapel Hill, 323 MacNider Hall, CB #7240, Chapel Hill, NC 27599-7240 USA; 4 Department of Health Behavior and Health Education, University of Michigan School of Public Health, 1415 Washington Heights, Ann Arbor, MI 48109-2029 USA; 5 Department of Health Policy and Management, Gillings School of Global Public Health, University of North Carolina at Chapel Hill, 170 Roseneau Hall, CB #7400, Chapel Hill, NC 27599-7400 USA

**Keywords:** Cardiovascular disease, Social and built environment, Geography, Health disparities, Qualitative methods

## Abstract

**Background:**

AA living in rural areas of the southeastern U.S. experience a disproportionate burden of cardiovascular disease (CVD) morbidity and mortality. Neighborhood environmental factors contribute to this disparity and may decrease the effectiveness of lifestyle interventions aimed at preventing CVD. Furthermore, the influence of neighborhood factors on AA CVD risk behaviors (i.e. physical activity) may be obscured by the use of researcher-defined neighborhoods and researcher-defined healthy and unhealthy places. The objective of this study was to elucidate the effects of neighborhood environments on AA CVD risk behaviors among AA adults who recently completed a lifestyle intervention. We specifically sought to identify AA adults’ self-perceived places of significance and their perceptions of how these places impact CVD risk behaviors including diet, physical activity and smoking.

**Methods:**

We conducted semi-structured interviews with AA adults (*N* = 26) living in two rural North Carolina counties (Edgecombe and Nash, North Carolina, USA). Participants were recruited from a community-based behavioral CVD risk reduction intervention. All had at least one risk factor for CVD. Participants identified significant places including where they spent the most time, meaningful places, and healthy and unhealthy places on local maps. Using these maps as a reference, participants described the impact of each location on their CVD risk behaviors. Data were transcribed verbatim and coded using NVivo 12.

**Results:**

The average age of participants was 63 (SD = 10) and 92% were female. Places participants defined as meaningful and places where they spent the most time included churches and relatives’ homes. Healthy places included gyms and parks. Unhealthy places included fast food restaurants and relatives’ homes where unhealthy food was served. Place influenced CVD risk behaviors in multiple ways including through degree of perceived control over the environment, emotional attachment and loneliness, caretaking responsibilities, social pressures and social support.

**Conclusions:**

As we seek to improve cardiovascular interventions for rural AA in the American South, it will be important to further assess the effect of significant places beyond place of residence. Strategies which leverage or modify behavioral influences within person-defined significant places may improve the reach and effectiveness of behavioral lifestyle interventions.

**Supplementary Information:**

The online version contains supplementary material available at 10.1186/s12889-021-12022-x.

## Background

Cardiovascular disease (CVD) is a leading cause of death in the U.S., with an especially high burden among African American (AA) adults in lower resourced, rural areas of the Southeast [1–3]. Cardiovascular disease interventions seek to equip individuals to make behavioral changes to improve their CVD risk behaviors. Recent studies have demonstrated that neighborhood-level factors may modify CVD intervention outcomes, suggesting that individuals actively attempting to change behaviors (e.g., eating healthier, being more active) may be helped or hindered by more or less supportive environments [4, 5]. Understanding how those at high risk for CVD experience and navigate their environments in relation to CVD risk behaviors is critically important to designing effective interventions to address CVD disparities [6, 7]. This work is particularly important among rural African Americans due to persistent disparities in CVD risk, morbidity and mortality [8, 9].

The influence of the physical environment cannot be treated as fixed and isolated, but rather, as dynamic and interrelated with how individuals experience and perceive each place alone and with others [10, 11]. Moreover, researcher determined “neighborhoods” used in much of the prior and current literature may not be concordant with how humans travel nor illuminate the diversity of experiences that occur within them [10, 12, 13]. Furthermore much of the existing research describing neighborhoods and health may not be as salient in rural communities that are largely dependent on automobile travel [14]. Person-defined significant places are therefore potentially more relevant than researcher-defined places for understanding environmental influences on CVD [15, 16]. Furthermore, an inductive qualitative approach may help us better understand the influences on health behaviors that exist within person-defined places [10, 17–19]. These influences may be particularly evident among individuals who are actively attempting behavior change.

The purpose of our study was to explore the perceptions of participants who recently completed a lifestyle intervention. Specifically, the objectives were to 1) identify places that were significant to African American adults, defined as places where they spent the most time, meaningful places, healthy or unhealthy places, and safe or unsafe places and 2) describe their perceptions of how these places influence their CVD risk behaviors following the intervention including dietary choices, physical activity, smoking and stress.

### Approach

#### Participants and setting

This study was performed immediately following the conclusion of an evidence-based lifestyle change intervention for the prevention of CVD, Heart Matters [20]. Heart Matters is a 12-month, behavioral lifestyle change intervention adapted from the PREMIER intervention [20]. Heart Matters aimed to improve behavioral and health outcomes including diet, physical activity, weight and blood pressure among AA adults with cardiovascular risk factors, and consisted of 14 group sessions and four individual sessions [20, 21]. A convenience sample of *n* = 26 participants from *N* = 72 participants in this intervention (response rate = 36%) were recruited from Nash and Edgecombe Counties, two of the most socioeconomically distressed counties in North Carolina [22]. This study is an ancillary study and was not a formal evaluation of the intervention.

All participants enrolled in the Heart Matters intervention were eligible to participate in the present study. Intervention inclusion criteria included being an AA adult with self-report of at least one cardiovascular risk factor: pre-diabetes or diabetes, hypertension, obesity, family history of early cardiovascular disease, prior diagnosis of cardiovascular disease. Individuals with active or unstable CVD or cognitive impairment that limited informed consent were excluded. Members of the study team verbally introduced the study and handed out fliers at 14 group intervention sessions held at churches, community centers and libraries. After participation, participants were provided with a gift card of $25. This study was approved by the Institutional Review Boards of participating universities.

## Methods

To permit person-defined environmental characterization, qualitative methods were chosen to capture community members’ experience of places significant to them [10]. Semi-structured one-on-one interviews occurred at locations that were convenient for participants, including churches, libraries and community centers. The five interviewers (three AA, two White) were college-educated females aged 20–40 living outside the study sites. Prior to conducting the interviews, all interviewers participated in a 2-h training led by a member of the team who is a doctoral-level qualitative research expert. During the training, researchers read the interview protocol and practiced skills for conducting semi-structured interviews.

Twenty-six semi-structured interviews were conducted from June to July 2018. Prior to the interview, participants provided written informed consent to an audio recorded interview. Participants were presented with three maps of varying scale: (a) entire town, (b) area 1–2 miles, and (c) 5–8 miles around the participant’s home. Participants interacted with the maps to promote open discourse and provide a visual reference point for in-depth discussion [23]. On each map, the location of the participant’s home was marked with a star along with commonly recognized landmarks such as the library, churches and retail locations. To identify participant’s significant places they were asked to mark 1–3 places on the map of their choice in each of the following categories: where they spend the *most time*, places that are *meaningful* to them, places that help them to be *healthy*, places that *keep them from being healthy*, *safe* places and *unsafe* places. We did not define the categories for the participants so that their answers would reflect their own conception of most time, meaningfulness, health, and safety. Participants were informed that they could mark one place with multiple categories. To determine how these places affected participants’ CVD risk behaviors the interviewer asked a series of questions about each of the places mentioned. (Supplementary file 1: “Qualitative Interview Guide”) Interviews were transcribed verbatim. Recordings were preserved and utilized to verify participant responses. Transcripts and the maps used by participants were imported into NVivo 12 software for qualitative analysis [24]. All transcripts were de-identified while maps containing address data were used to confirm specific locations. Transcripts, recordings, and maps were stored on a secure server throughout the duration of the study.

The qualitative analysis team (a physician, an epidemiologist, and a geographer; two AA, one white) created the codebook and coded the transcripts to identify salient themes which represented answers to the research questions. Two of the researchers were experienced with qualitative research. All three participated in a training on qualitative analysis and coding led by a doctoral-level team member with expertise in qualitative analysis.

**Fig. 1:**
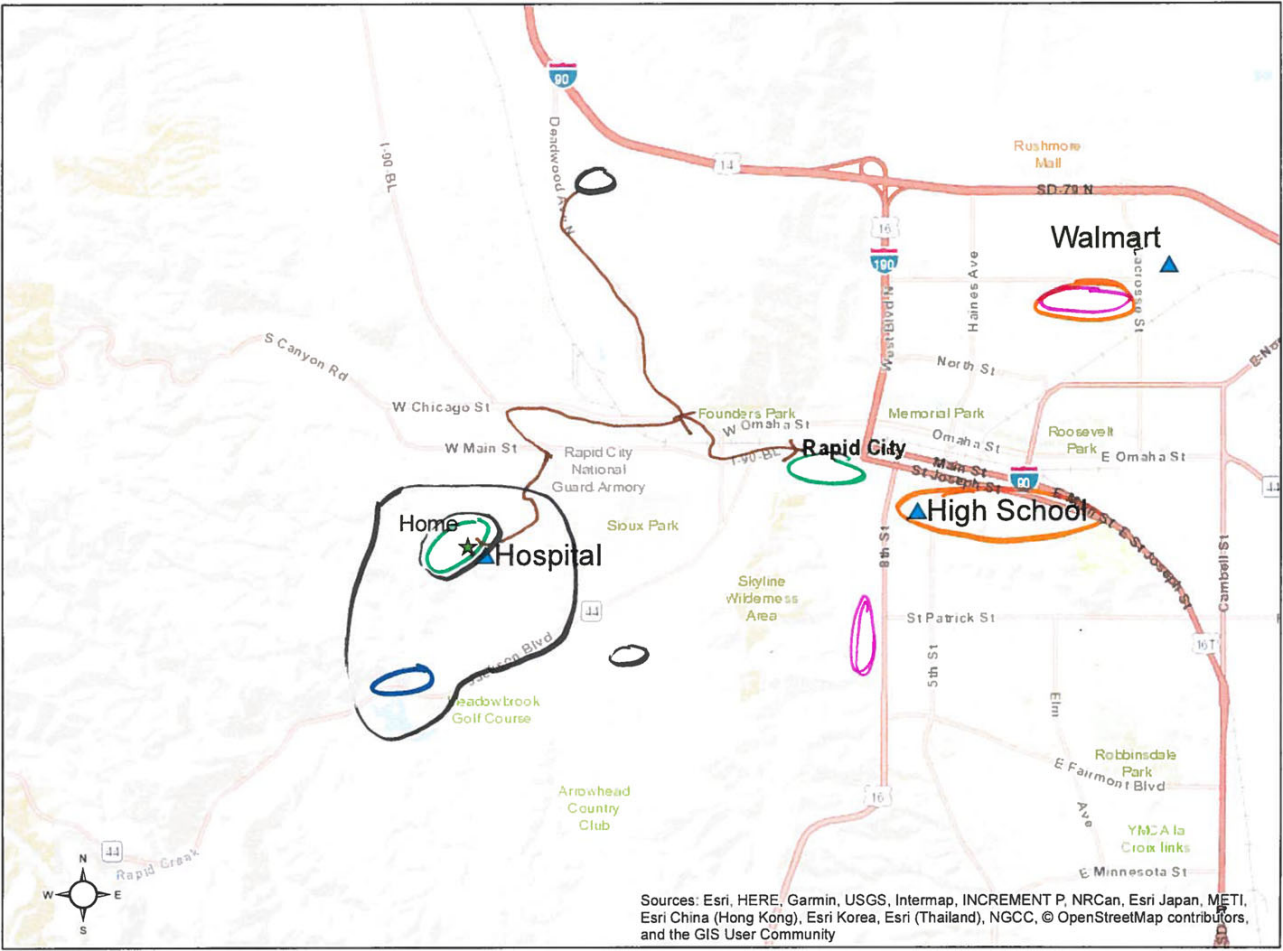
Mock Participant Map

First, the analytic team reviewed all transcripts and maps to immerse themselves in the data. The team identified three, data-rich transcripts to develop the codebook. Using these transcripts, each member of the analytic team created a separate draft codebook. The draft codebooks were then discussed amongst the analytic team until a consensus codebook was created. Each transcript was subsequently coded in duplicate and adjudicated by consensus. Participants’ maps were used to clarify locations and location categories (Fig. 1: “Mock Participant Map”). Using an inductive approach consistent with the constant comparative method, the analysis post-coding consensus was focused on developing emerging themes, or patterns, across codes [25, 26]. During the analysis, we also assessed whether new codes or themes were emerging and found the *n* = 26 sample was more than ample to achieve saturation. After creating an initial summary of findings, a separate member of the research team (a white, female doctoral-level researcher) who was not involved in data collection or the initial analysis served as an external auditor. She participated in analytic discussions, reviewed the data and initial interpretation separately, and provided feedback to the analysts to support the rigor of the data interpretation and to minimize analysts’ biases. Prior to finalizing data interpretation, results of the analysis were presented to key community stakeholders. All stakeholders were AA adults who lived in Edgecombe or Nash counties. The results presented herein were affirmed by key stakeholders and the study team.

### Application of four tenets of trustworthiness

To ensure the rigor of our study we addressed the credibility, dependability, confirmability and transferability in the following ways [27, 28]. To ensure that results were credible all interviewers were trained in semi-structured interviewing. During the analytic process we conducted negative case analysis- seeking instances which contradicted our interpretation of the data [29]. For dependability and confirmability we employed weekly meetings of the analytic team, a detailed study protocol and an external individual conducted an audit of data analyses and interpretation. Also we presented our study results to key stakeholders who affirmed the results of the study. For transferability we describe the context in which the data was collected (AA older adults with cardiovascular risk factors who recently completed a behavioral lifestyle intervention in the rural southeastern, U.S.) such that our study conclusions could be tested in a similar or alternative context.

## Results

### Participant characteristics

There were 26 African American participants, of whom, two were male (Table 1). Ten participants were married; the rest were single or widowed. Participants’ level of education varied: 38% had a high school education or less, 35% had attended college or technical school and 19% had a college or graduate degree. The average age of participants was 62 (SD = 11).

**Table 1:** Participant Characteristics

Participant Characteristics (***N*** = 26)	% (N)
**Gender**	
Male	8 (2)
Female	92 (24)
**Marital Status**	
Married	42 (10)
Single	33 (8)
Widowed	25 (6)
Missing	8 (2)
**Education**	
High school or less than high school	38 (10)
Technical School/Some College	35 (9)
College or graduate degree	19 (5)
Missing	8 (2)
	**Mean (SD)**
Age	62 (11)

### Significant places reported by participants

Table 2 provides a summary of the specific types of significant places noted and how frequently they were referenced in the six categories: (1) most time, (2) meaningful, (3) healthy, (4) unhealthy, (5) safe, and (6) unsafe places. A brief summary is provided below to provide context for the qualitative discussion of how participants perceived and experienced these significant places in relation to CVD risk behaviors.

**Table 2:** Participant References to Significant Places (*N* = 26)

	Home		Church		Retail		Parks/Rec		Home of Family/Friend		Work		Library/Community Center		Healthcare		Restaurant		Other		Total References
	N	%	N	%	N	%	N	%	N	%	N	%	N	%	N	%	N	%	N	%	
Most time	23	31	13	17	10	13	7	9	7	9	6	8	5	7	3	4	–		1	1	75
Meaningful	18	25	15	21	6	8	8	11	8	11	3	4	10	14	1	1	–		2	3	71
Safe	24	49	12	25	–	–	1	2	3	6	3	6	4	8	–	–	–	–	2	4	49
Unsafe	1	6	1	6	5	29	3	18	1	6	1	6	–	–	–	–	1	6	4	24	17
Healthy	11	18	6	10	6	10	13	22	3	5	3	5	6	10	5	8	5	8	2	3	60
Unhealthy	6	15	–	–	7	18	3	8	2	5	3	8	–	–	–	–	19	48	–	–	40
Total	83		47		34		35		24		19		25		9		25		11		312

#### Most time and meaningful places

Home was the most common place cited where participants spent the most time; only three participants did not report home within this category. Church and retail locations (e.g. grocery stores) were also commonly mentioned places where participants spent most of their time, representing 17 and 13% of reported locations, respectively.

Home and church were also the most commonly reported meaningful places, representing 25 and 21% of reported meaningful locations respectively. Participants stated that places were meaningful for a variety of reasons. Most commonly, places were described as meaningful because of the people who were present, memories of family or friends, or the sense of peace and relaxation participants felt within them.

#### Safe and unsafe places

Participants largely described home and church as safe places. Half of participants were unable to name a specific place that they deemed unsafe. When identified, the most commonly reported unsafe locations were retail locations. Most participants who named an unsafe place explained that they chose to label the place unsafe due to the potential for crime because “you don’t know who’s in there” or “anybody can walk in and do anything” rather than describing specific experiences that made them feel unsafe.

#### Healthy and unhealthy places

Most participants were able to identify locations that they felt supported their health. Parks and recreation facilities were most commonly reported (22% of reported healthy locations), followed by home (18%). Participants identified far fewer unhealthy location types, but nearly half of those were restaurants. Notably, four participants listed the same place as both healthy and unhealthy and indicated contrasting reasons for their categorization. For example, one participant noted their worksite offered support in the form of blood pressure checks (healthy) but also was a source of stress (unhealthy).

### Perceptions related to CVD risk behaviors within significant places

The following describes eight themes from the data related to the influence of place on CVD risk behaviors after the 1 year behavioral lifestyle intervention. The first three themes highlight influences which appeared to occur in specific significant locations. The remainder relate to social interactions, emotional responses, and commitments that occur across a variety of significant locations.

#### Perceived control supports positive CVD risk behaviors within the home

For some participants, home was a location where they felt in control of their environment and able to make positive decisions about CVD risk behaviors, particularly related to diet and mental health. Within their homes, participants often focused on the ability to control food preparation for themselves as well as preparing meals for family and friends (Table 3). For example, one participant noted:

“When anybody comes to my house I let them know that I don't have salt [ … ] So I feel healthier at home because I'm able to purchase the things I learn about at [the intervention],”

**Table 3:** Example Quotations: Perceptions and Experiences Related to Cardiovascular Disease Risk Behaviors Within Significant Places (*N* = 26)

*1. Perceived control supports positive cardiovascular disease risk behaviors within the home*	“I feel like I’m in charge of my whole whatever I do, you know. And I’m – I’m comfortable there because, um, I can relax and, um, the way that I want.”
	“You know what you [can] stand to eat and what you can’t. So, you know, when you go to a restaurant, you can’t tell them, oh, look at all this salt, you know. But yeah, you could fix your food like you want at home, and, you know, eat healthy, you don’t have to, you know, eat too much like that.”
*2. Living alone influences cardiovascular disease risk behaviors within the home*	“A majority of the time, because I live alone so I don’t cook all the time like I did when I was raising my family, I might just pick up something and take it home.”
	“A lot of times with just being me in the household, it’s cheaper just to pick up something quick and convenient than it is to try cook.”
	“Well, I don’t cook as much because, you know, it’s just me. But I do watch what I do eat and stuff like that. Sometimes I go off a little, but it’s basic, yeah.”
	“But when I go back into the house – and sometimes I get lonely – I don’t tell my family that because they would be there all the time. But I like being home, of course. Everybody likes being home. But sometimes, uh, I get lonesome.
*3. Safe environments which reduce stress positively influence cardiovascular disease risk behaviors in parks and recreational facilities*	“Well [walking in the park is] such a habit now. It [local park]– it’s serene, it’s serenity because when you’re walking you have time to think, relax. And so that’s part of my serenity place out there.”
	“I don’t necessarily know these people by name, but I know who they are and know what type of car they drive. I know what time they’re going to get there [the park] … It’s just a nice place to be.”
	“… when I walk it takes a lot of stuff away from you. Your mind just go clear because you ain’t thinking about it.”
*4. Caretaking responsibilities influence cardiovascular disease risk behaviors across a variety of places*	“But now, so since my daughter’s working up here to the Social Service in Tarboro, she don’t get off until 5:00, so I had to keep my granddaughter.”
	“And, um, you know, after she [daughter] died, her – my grandchildren was at my house a lot ‘cause my son-in-law, he had to go to work”
	“And when it comes to medical in my house, it’s me. I’m the girl that do the pill trays. I’m the girl that takes everyone [sisters and father] to the doctor. I’m the one that makes sure everybody is ok when it comes to the medical.”
	“Once I leave my client, I always go check on her [my mom] because they’re on the same side of town and everything. And, um, she’s dealing with Alzheimer’s. And everything. So I’ll spend like an hour, sometimes two hours, with her. And, um, see what she needs.”
*5. Social pressures negatively influence cardiovascular disease risk behaviors across a variety of places*	“If I was going to choose any place to go I would not choose a diner. [I go] just because my husband likes to go over there.”
	“Last Sunday – yeah, last Sunday, my parents and my cousin, we stopped after the church thing. We, um, we ordered us a meal and sat down at the table and ate. Ate them fries, boy, I’ll tell you, I think I drank two cups of tea because the fries were so salty. So I had to go tell them, I was like, look, right here, can’t you see all this swelling I have here? You gonna kill a sister.”
	“They’re not going to have a lot of healthy things ‘cause most churches, well a lot of churches don’t eat healthy.”
*6. Social support positively influences cardiovascular disease risk behaviors across a variety of places*	“Well, at church, we have a group of ladies, we have lunch together […] we always try to eat healthy foods and we talk about the foods that we eat. So those things is encouragement, you know.”
	“Like I was saying about the community center, when you’re down there with those ladies, always healthy all the way … Those ladies don’t argue with them, go with the flow.”
	“[The senior center] that’s a safe place for me, ‘cause, you know, I know a lot of people that’s there, and they’re old, ‘cause you got to be 55 and older, and so it’s comfortable to me.”
*7. Emotional attachment, and tradition negatively influence cardiovascular disease risk behaviors across a variety of places*	“We have – our family has – at least one of us, my mama and my sister, my nieces, my children, we get together once a month. And you either cook or you take the family out. So this Sunday, uh, is my – well July is my mama’s month, and she likes Golden Corral. She’d rather go there. And of all the buffets I’d rather do there too. But the family loves Grandson’s, so that’s where they want to go, and she’s given in to them. So that’s the only reason I go.”
	“[The Elks Lodge], it’s like being at home [because] they treat you like at home.”
	“[My aunt and uncle’s house] feel like home. Feel like my momma’s house.”
*8. Participants influence the cardiovascular disease risk behaviors of others across a variety of locations*	“Well what I had done is recruit – because I do have two people that are a part of it – not part of our session but that are part of Heart Matters. And the foods that they introduce us to, I’ll share that with the people in the office and – well in our office and in other offices.”
	“I have a friend and a cousin that we talk to, […] she’ll say, “What did you learn at Heart Matters?” you know, so I’m helping her– and we’re spreading the word.”
	Participant: “And I tell them how to eat.”
	Interviewer:” So you’re trying to tell them how to eat and kind of what grease to use, okay.”
	Participant: “Yeah. Mm-hmm. And all that salt my grandson love it. And they saw lots of butter […]”
	Interviewer: “Okay. And are those things that you learned in Heart Matters? Are you –”
	Participant: “Spreading around. ‘Cause I told him, I said, “You want to live longer, you got to eat healthy.” So they’re learning now.”

Home also contributed to the mental health of participants as a location where many felt comfortable, stress-free, and safe because of the control they had over their surroundings.

“I just feel safe in the house. I say because [it’s] my house and I know what’s going on in my house.”

#### Living alone influences CVD risk behaviors within the home

A subset of participants reported living alone as they described their home environment. Living alone influenced diet, physical activity, smoking, and mental health. For example, some participants found living alone to be a barrier to healthy eating after the intervention (Table 3). As one participant explained, she often skipped breakfast and lunch:

“My mom … she like, ‘Girl, cook the food and sit down and eat.’ [but] you know when you're sitting in the house, physically alone [ … ] it's hard to eat alone. The food is just not good when ya gotta sit there [alone].”

Another noted that her frequent purchase of TV dinners was due to her frustration of trying to cook for one without wasting food:

“I have a tendency a lot of times when I do cook, that I, I get upset because it's so much food wasted and everything [ … ] But it's just, um, nerve-wracking knowing you spend money and you're wasting money like that.”

Living alone also led to boredom and loneliness, which participants connected to unhealthy behaviors like smoking and excessive screen time (Table 3). One participant, who lived alone with minimal access to transportation, said that she was often bored at home and was struggling to follow intervention guidelines to quit smoking:

“Sitting at home. If I had a car, I probably wouldn't smoke that much, because I’d be on the go all the time to visit my people and family and stuff like that. But sitting at home, you’re gonna smoke.”

At the same time, participants also perceived positive aspects of living alone, often because of perceived control, as described in the prior section. Living alone also had positive influences emotionally for a handful of participants, one of whom described her contentment when she is alone at home.

“That's why I just spend a lot of time alone and whatnot. But it doesn't, you know, like some people, it affect them. For me, I'm content.”

#### Safe environments which reduce stress positively influence cardiovascular disease risk behaviors in parks and recreational facilities

Healthy places for participants included the YMCA, the senior center, and the local hospital’s track. They appreciated the availability of these resources in the community. For example, one noted that “[the hospital track] it’s inviting, because they’re always improving it … a lot of people can go out there and walk, you know. And don’t be exposed to everyday traffic.” Others spoke of using these locations to reduce stress.

“I feel very comfortable, relaxed [at the YMCA] … after I leave, I feel like a different person. You know, I – it just feel– like stress-free.

#### Caretaking responsibilities influence CVD behaviors across a variety of places

Caretaking was a key social influence on participants’ health behaviors by causing stress, being a significant time commitment, and limiting participants’ ability to follow intervention guidelines (Table 3). Participants described being a caretaker for a variety of family members including grandchildren, elderly parents, aunts and uncles in participants’ homes or the homes of relatives. Caretaking was often a stressor, which led to health choices that participants perceived as negative. One participant, whose father with dementia recently moved in with her described her experience:

“So that’s another set of stressor all by itself [father moving in] and so my problem now is how to deal with the stress without eating. ”

She went on to describe her desire to join the YMCA but she was unable to do so because she couldn’t leave her father alone. She and several other participants also mentioned that they were often tempted to eat the unhealthy foods they purchased from restaurants or stores at the request of individuals they cared for.

#### Social pressures negatively influence CVD risk behaviors across a variety of places

In addition to caretaking, social pressures influenced participants CVD risk behaviors in a variety of places. Some participants who lived with their families described how members of the family contributed to unhealthy choices in their homes (Table 3). Some family members were resistant to the dietary modifications participants learned during the intervention, making it difficult to implement them at home. One participant related,

“Well my husband, he don’t like for me to go to the grocery store, [because] he say he ain’t on no diet [laughter]. [ … ] So I buy a lot of boneless chicken, he be like, don’t nobody want no boneless chicken breast [ … ].’”

Similarly, others noted that factors they could not control such as media and their neighbors made their home environment less supportive of healthy CVD risk behaviors. One participant said,

“A commercial come on the TV where somebody talking about [smoking], or you're sitting there looking at the TV, and I look over to the door [where I] can see outside [ … ] Talking about quitting smoking. But there they go. [My neighbor] light him a cigarette.”

Outside their homes, participants’ family and friends influenced their food choices via their preference for specific restaurants and meals at family gatherings (Table 3). In these cases, the participant considered the restaurants unhealthy places, and described going to these places primarily due to social pressure from family and friends.

“It's a big buffet place. Mm-hmm. And my family loves to go there and I will always say, ‘I don't want to go,’ because I don't like a lot of food spread out. You know, I – I don't like buffets period. But I always go to please them.”

Even when participants described being able to use the skills they developed in the intervention to make healthy choices in the restaurants they described, they still considered those restaurants to be unhealthy places.

“So what I did was because of the restaurants that I like, um, on the strip I learned how to go and if my family still wanted to go or whatever I would look at the menu. And get whatever was going to be good for me that was still going to be good that I liked.”

The preferences and habits of coworkers also influenced participants diets at their workplaces:

“ … they eat all the time [at work] and then we eat unhealthy food all the time [ … ] they selling something all the time, and it's not healthy. Then, we stay — we like, right across the street from Maxway and a lot of them go over there and get snacks and stuff [ … ] So you have to really, really be strong at work.”

Finally, church was a frequently mentioned significant place where participants described social pressures from family and friends (Table 3).

“They [prepare] a full meal. You know how church people cook. And, uh, you know, you’re exposed to it if you want it [ … ] collards and, you know, things like that.”

#### Social support positively influences CVD risk behaviors across a variety of places

In contrast to negative social pressures, participants also described social support for healthy behaviors across multiple places including parks and recreation facilities and churches (Table 3). This was most frequently evident in participants’ descriptions of the places where they engaged in physical activity. For example, familiarity with others who walked in local parks encouraged participants to exercise. Participants also felt local recreation facilities supported healthy behaviors because there was peer support in a safe, welcoming environment:

“ … you have a group in there that we all encourage each other and plus we have instructors that make it [aerobics] exciting … [ … ] I feel comfortable. I guess the environment is … It's nonjudgmental.”

Some participants attended churches where the leadership and members were committed to a healthy lifestyle resulting in healthier food options offered at church social events. There were also church-based walking and exercise groups. The social influence of church on participants’ health behaviors resulted from shared accountability, particularly when others at the church were striving to adhere to a healthy diet.

“We always, you know, try to remind each other to not use salt, don’'t drink sodas or sweet teas in here.”

“They make sure – the pastor try to make sure everybody eat right. They cook [and] they make sure it's the right food, not a whole lot of greasy food and junk food.”

#### Emotional attachment and tradition negatively influence CVD risk behaviors across a variety of places

Many participants described going to self-defined unhealthy places because the physical or social environmental attributes of the location reminded them of “home” (Table 3). The sense of emotional attachment to place appeared to drive participants’ decisions to visit specific places and engage in specific CVD risk behaviors as this participant illustrates describing her decision to dine at a restaurant she deemed unhealthy:

“It looks like my grandmama’s kitchen … because it’s so full of stuff that she cooked that I grew up on. I mean cabbage, collards, yams, baked spaghetti, chicken, grilled or fried pork chops. So I like that because it’s so down home to me.”

Others discussed how family traditions surrounding special events and holidays influenced their decisions to visit specific places and make behavioral choices which influence CVD risk.

“Well, we go there about – we meet – meet every holiday at one [family member’s] house or the other. And then we do all this cooking and, you know, they ain't learn how to cook like I cook yet. So we meet then and that's when we eat the unhealthy food.”

#### Participants influence the CVD risk behaviors of others across a variety of locations

Not only did participants experience social pressures and support from others, they also intentionally tried to influence the health behaviors of others in various places, including homes, workplaces, and churches (Table 3). For example, one participant who worked as a Certified Nursing Assistant described reducing her client’s salt intake and another discussed how she shared new foods introduced through the intervention with her co-workers.

Participants also attempted to make lifestyle changes for themselves and their families based on what they learned in the intervention within their homes. One participant lived with her two sisters and her elderly father, whom they cared for. She talked about changes she implemented based on her experience in the CVD prevention intervention.

“You know and because I do most of the cooking it was easy for my family to transition to whatever I thought was good. They didn’t fight me on it [ … ] and the snacks, um, it helped with me making sure my daddy was eating healthy snacks.”

## Discussion

Across a broad array of places participants shared several ways that the significant places in their lives influence their CVD risk behaviors including social pressures, caretaking responsibilities and the availability of recreational amenities. Our findings are consistent with prior studies that have demonstrated that health choices are the result of interactions between individual beliefs, peer influence and opportunities afforded by the communities in which individuals live [30–32]. Our study expands existing literature in several important ways. First, we focus on AA living in rural counties in the southeastern U.S., where CVD risk is known to be in the highest in the country, while the majority of similarly focused literature involves participants in urban centers [30, 32]. Second, the timing of our study, following a CVD prevention intervention, provides an important window into how environmental influences shape participants’ responses to a lifestyle intervention and their ongoing efforts at chronic management of cardiovascular risk. Third, our results reveal that researcher-defined healthy and unhealthy places, measured via distance or density around participants’ homes, are not likely to capture the varied place-based factors which may influence intervention outcomes or the sustainability of intervention effects [15, 16]. Cumulatively, our work indicates that behavioral interventions which aim to promote healthy lifestyle choices may prove more effective when developed with an understanding of the perceived physical and social behavioral influences within person-defined significant places [11].

A clear pattern across most of the participants was that significant places, particularly the social influences within these places, served to reinforce healthy behavior change through accountability or the presence of others who supportive of healthy behavior change. Conversely, negative social pressures within significant places created barriers to healthy behavior change. While the significance of social influences on health behaviors has been widely described, participants’ place-based descriptions of these processes is noteworthy for the reach and implementation of behavioral interventions [33, 34]. For example, participants’ significant places may represent opportunities to disseminate interventions beyond the participant themselves to their families, friends and coworkers. Similarly, interventions which address the social pressures within person-defined significant places such as work or church in addition to individual-level behavior change strategies may lead to improved intervention outcomes and sustainability of intervention effects [35, 36]. For AA adults in rural settings, partnering with leaders and influential individuals within key significant places such as churches and workplaces while using these places together as intervention settings, may be a means of addressing negative social pressures and leveraging place to improve CVD risk behaviors. Our data specifically indicate the importance of home, church and retail locations. These places may represent ideal targets for multilevel interventions. One example of this approach would be an intervention which includes the following components: 1) online tools to promote healthy choices at home 2) church-based lifestyle groups who provide social support and accountability for healthy choices and 3) an agreement with local grocery stores to display flyers as visual cues to reinforce healthy choices. Joint use agreements are policy-level tools which may facilitate this type of intervention [37]. Multilevel interventions which leverage individual, interpersonal, community and policy factors reflect the socio-ecological model of disease and have been proposed as a key method for improving health disparities [38].

Our study highlights living alone as a specific home context that merits distinct consideration, particularly during the COVID-19 pandemic which has led to increased focus on the potential health vulnerability of older adults who live alone [39, 40]. Living alone is associated with multiple health outcomes including risk of a major adverse cardiac event, depression and unplanned hospitalization [41–45]. The increased risk of a major adverse cardiac event may be due to dietary factors, as eating alone can lead to reluctance to prepare meals and reduced diversity of meals, as described by some of our participants [41]. Importantly, some participants who lived alone ascribed some advantages to living alone such as greater control of their diet and health behaviors, and a sense of peace and well-being. These potentially positive attributes of living alone allude to the presence of factors which may be protective among adults living alone [43, 46]. Describing risk and protective factors among individuals living alone may yield a tailored approach to CVD interventions, such as promoting simplified recipes with fewer servings and enhancing participants’ social support for healthy behaviors within communities.

Caretaking was an emergent theme which influenced participants’ diet, physical activity and stress across multiple significant places. While much is known about marriage and family relationships and their influence on health behaviors, less is known about caretaking relationships and their effect on CVD risk behaviors [47, 48]. While caretaking was often described as a negative influence on CVD risk behaviors in our cohort of participants, caretaker contexts were also opportunities to disseminate the lessons learned from the lifestyle intervention. Our findings support increased emphasis on the development of CVD interventions that address the stress of caretaking and leverage caretaking relationships as opportunities for dissemination, when appropriate. This type of intervention would be particularly well-suited to older African American women, who tend to bear a large burden of caretaking responsibility [49].

Our findings should be considered in the context of several limitations. These data were collected at the conclusion of a CVD intervention, which may have increased the potential for social desirability bias, as participants may have preferentially shared their successes and accomplishments. Additionally, our sample was largely comprised of AA women in the rural southeastern U.S. and may not be generalizable to other demographic groups such as men and younger AA adults without CVD risk factors. Men made up a relatively small portion of participants of the larger Heart Matters intervention study, thus there were few male participants in the present study. Strengths of our study included the use of maps as visual prompts to promote the identification of a variety of significant places. Also, our use of a multidisciplinary team from outside the study area allowed for an openness to participants’ interpretations and descriptions of places unencumbered by prior knowledge of the places described. Finally, our use of an additional team member to audit data analyses and interpretation and our incorporation of key stakeholder comments as part of the interpretive process strengthen the validity and reliability of our findings.

## Conclusion

Participants in our study described the influences of the significant places in their lives on their CVD risk behaviors following a year-long lifestyle intervention. The impact of these significant places on CVD risk behaviors was largely a product of the social influences present there, the perceived availability of health-promoting food, amenities and activities and emotional attachment to these spaces. These findings highlight several important considerations regarding the role of place in the development and implementation of lifestyle interventions. Understanding the influence of significant places may yield opportunities to tailor interventions to participants’ physical and social environments.

## Supplementary Information

**Additional file 1.** Electronic Supplementary Materials: Qualitative Interview Guide.

## Data Availability

The de-identified versions of the datasets used and/or analysed during the current study are available from the corresponding author on reasonable request.

## Abbreviations

CVD: Cardiovascular disease
AA: African American
