# Supplementary material for: The significant places of African American adults and their perceived influence on cardiovascular disease risk behaviors
Source: BMC Public Health. 2021 Nov 5;21:2018. doi: 10.1186/s12889-021-12022-x (PMC8570769; doi:10.1186/s12889-021-12022-x)
Supplement: Supplementary file 1 — Additional file 1. Electronic Supplementary Materials: Qualitative Interview Guide. [file 12889_2021_12022_MOESM1_ESM.docx]

**Interview guide**

**Map/orientation:**

Great, let’s begin the interview. [Bring out the participant’s map] This is a map of Nash and Edgecombe counties.

**Option #1 [If the participant’s address was available from Heart Matters, it will be marked as a star on the map].** Based on Heart Matters records, your address is here [show anchor at their home address] – is this correct? [If yes, proceed. If no, ask them to mark their home on the map].

**Option #2 [If the participant’s address was missing from Heart Matters data, there will not be any address marked].** Can you please mark on this map, where you currently live? [Ask them to mark it with a star]

***Questions about places***

1. I’m going to ask you to please mark different types of places on the map and then ask a series of questions about each:

[As you ask them to mark these places, ask them to use the indicated color for each category]

- 1. In black, mark 1-3 places where you spend the *most time* (i.e., the greatest portion of your overall time in a week)
     1. Ask #2 questions about these places
  2. In blue, 1-3 places that are most *meaningful* to you (these may or may not be the same as *most time* places)
     1. Ask #2, #3, and #4 questions about these places
  3. In green, 1-3 places that *help you to be healthy* (these may or may not be the same as the places you already identified)
     1. Ask #2 questions about these places
  4. In red, 1-3 places that *keep you from being healthy* (these may or may not be the same as the places you already identified
     1. Ask #2 & #5 questions about these places
  5. Finally, in orange, please mark (if it exists) a place on the map where you feel the *safest*? The *least safe*?
     1. Ask #2 questions about these places

1. For *each* of these places:
   1. How often are you at this place? (e.g., every day for most the day, once a week for a few hours, etc.)
   2. What does this place look and feel like to you?
   3. How do you feel when you are in this place?
   4. What do you do in this place?
      1. Probe: Are there certain people that you often interact with in this place? If so, who are they and what do you do together in these places?
      2. Probe: What health-related behaviors happen in this place? For example, do you eat, exercise, smoke cigarettes, etc. in these places?
      3. Probe: As you have been involved in Heart Matters over the past year, have you noticed anything about these places that were either helpful or unhelpful to living a healthier lifestyle?
2. For *meaningful* spaces:
   1. What about this space makes it important and meaningful to you?
   2. (if they don’t mention people) Are there any people who make this space important or meaningful?
3. If *meaningful* spaces are different than the *most time* spaces:
   1. How is this place different than the spaces you spend the most time in?
4. For places that *help/hinder* *health*:
   1. What is similar and different about the places that help versus the places that keep you from being healthy?

***Questions about a typical day***

1. Now, I would like you to tell me a story about a typical day for you over about the last 6 months. You can use the map to tell me about *where you go* and *what you do* in these places. Please feel free to draw arrows/lines and write notes on the map as you tell me your story.
   1. Probes (Transportation):
      1. How do you travel from place to place (walking, bus, driving own car)?
      2. Do you travel from place to place with anyone?
   2. Probes (Eating Behaviors):
      1. Where do you eat most of your meals (at home, work cafeterias, eat-in or fast food restaurants)?
      2. When you eat at home, how often do you eat home-cooked versus take-out or delivery?
      3. Which stores do you most buy food from (groceries and restaurants)?
      4. What are your favorite places to get food?
      5. Do you eat your meals with anyone?
   3. Probes (Physical Activity Behaviors):
      1. Do you ‘exercise’? If so, where do you go to exercise (i.e., stay at home, neighborhood, gym, etc.)?
         1. (if they go somewhere) How do you get to the places you exercise?
      2. Do you exercise with anyone?

***Final Questions***

1. Can you draw a circle around the area that you consider “your neighborhood”? How would you categorize your own neighborhood in terms of urban or rural - is it either of those or something different?

*Those are all the questions I have for you. Is there anything else that you would like to share with me about your experience with Heart Matters or about the places you live, work, and socialize?*

*Thank you so much for participating in this project and sharing with us about your experiences. Do you have any questions for me?*
